# Child and adolescent mental health service, Terengganu, Malaysia: how we are thinking about making a difference

**DOI:** 10.1080/17571472.2018.1484333

**Published:** 2018-07-09

**Authors:** Rahima Dahlan, Mohd Nizam Abd Ghani, Rosliza Yahaya, Tuan Saripah Tuan Hadi

**Affiliations:** a Faculty of Medicine, University Sultan Zainal Abidin, Kuala Terengganu, Malaysia; b Department of Psychiatry and Mental Health, Hospital Sultanah Nur Zahirah, Kuala Terengganu, Malaysia

## Why this matters to me

To effectively deliver child and adolescent mental health (CAMH) services in resource-constrained settings, it is vital to be smart in utilizing such resources efficiently, and this requires strategic planning. Of paramount importance is in-service training of primary care professionals aimed to consolidate existing knowledge and employ evidence-based strategies to manage child mental health care and to successfully integrate further these services into other mainstream healthcare facilities.

The implementation of CAMH services in Terengganu has served to facilitate numerous improvements such as promoting and increasing awareness of young people with mental health problems within their general practices. Furthermore, planning and implementing fully integrated child mental health service in Terengganu and the East Coast of Malaysia has served to enhance our understanding and yielded valuable insights which include:

• Enthusiasm from health professionals/ CAMHS team lead by a child psychiatrist and CAMHS team as the service provider is a key determinant of sustainability
• Perseverance and patience are the salient qualities necessary to secure commitments and resources from relevant authorities
• Liaising effectively and cultivating cooperative and communicative networks between primary care professionals, educators, child-care and social care professionals, as well as non-governmental organisations is essential for effective service network
• In-service education and training as well as ‘task-shifting’ methods to optimise human resource development can be potent strategies to sustain CAMH service in limited resource environments

The establishment of CAMH services in Terengganu and the East Coast of Malaysia has resulted in better skills in assessing and managing young people with mental health problems by medical practitioners and mental health workers. It has had a positive impact on young people with mental health issues and their families around the East Coast and particularly in Terengganu.

Child psychiatry services in Terengganu, Malaysia were initiated in 1997 as part of services provided by the department of Hospital Sultanah Nur Zahirah Hospital (HSNZ) which has been gazetted to manage psychiatric patients in Terengganu since early 70’s. The service was led initially by a general psychiatrist who has a deep interest in working with young people with mental health problems. A specific day was allocated for children and adolescents with mental health issues to attend the general psychiatric clinic.

The service development has progressed with the integration of child psychiatric unit specially designed and included during the planning of Day Treatment Complex in the 8th Malaysian Plan. This complex which also housed the psychiatric clinic was finally completed in April 2003. Sadly, the service was temporarily discontinued when the specialist was transferred to serve a different hospital. All children and adolescent patients were then relocated to the general adult clinic to be managed by medical officers and general psychiatrist.

In December 2004, the child psychiatry clinic was re-established and re-branded with a new name Child and Adolescent Mental Health service (CAMHS) and managed by a child and adolescent psychiatrist who was posted to the hospital. The CAMHS was moved to the existing Day Treatment Complex. There were, however, multiple challenges faced during the initial implementation of CAMH service. Among those, we encountered various constraints involving inadequate funding to provide effective training and crucial posts to set up a multidisciplinary team to facilitate the assessment and management of mental health needs of children and adolescents. Inadequate support received including a vague understanding to allocate appropriate staff namely occupational and speech therapists, clinical psychologist/counsellor dampened the proper assessment and effective interventions upon arriving at the diagnosis. It was more of ‘a one-man show’ type of approach; i.e. existing occupational therapist focused mainly on the physical deformities while speech therapists are based in the ORL department; primary health care providers were not managing the cases adequately.

## One thing we are doing to make a difference

Since 2005, great efforts were made. A series of training courses and workshops were carried out both by the department and by other agencies. Specific issues were identified and funding was appropriately explored to materialise such plans. The primary focus was on improving knowledge and useful skills among mental health practitioners in the care of children and adolescent mental health patients. We have:

• Encouraged more specialists to subspecialise in child psychiatry with ongoing training of Masters in child psychiatry as well as a training base for those planning to subspecialize in child psychiatry abroad
• Provided an opportunity for staff to further their study or doing an attachment in child and adolescent mental health programmes and activities
• Made sure that all training modules, guidelines and protocol on child and adolescent mental health have been made available to all schools, primary care clinics and communities.
• Collaborated with the Education Department in organising courses and workshops related to mental health for school children and teachers in Terengganu.

## Data or anecdotes about success so far

Despite such constraints and being the only CAMH service in Terengganu, the team has endeavoured to provide an efficient service that will give an impact to the state of Terengganu and East coast. As demonstrated in Table 1, between 2008 and 2018, in-service education and training for human resource development has been done throughout the years. Gradually, more primary care professionals and allied health have been trained on children mental health issues. In addition to the on-going training we have held awareness raising activities in child and adolescent mental health, been involved in research and development groups and conducted training modules national and locally every year since 2006 (Table 2).

**Table 1.** CAMHS: In-service education and training for human resource development each year between 2008 and 2018.

Focus multidisciplinary team	Training	Number of participants involved
Sub-speciality child and adolescent psychiatry training	Since 2017	2 trainees
Child and Adolescent psychiatry training for Master in Psychiatry program	2013 (4-months)	2
Child and adolescent psychiatry training 1^.^3r Master in Family medicine program	2012–2013 (2- months)	2
Primary health care professionals (Family Medicine Specialist& Medical Officers)	2/year (2008–2018)	50
Paramedics, allied health practitioners (Speech and occupational therapist) & social workers	2/year (2008–2018)I	50
Allied health practitioners (Speech, Occupational therapist)	2/year (2008–2018)	10
Hospital Continuous Medical Education (CME)	2/year (2008–2018)	80–100
Department CME	3/year (2008–2018)	15–20

**Table 2.** Involvement in the development of training modules, research, specific guidelines and awareness raising activities national and local between 2006 and 2017.

Planning an effective service for children and adolescents with specific learning disabilities in Malaysia
National level training on using the Manual on Managing Mental Health problems among adolescent for primary health care providers in for Northern: East coast; Central; Southern and East Malaysia zones, (WHO/KKM development group)
Clinical Practice Guideline on ADHD (MOH development group
Research on Malay validation of Children’s Depression inventory (CDI) and Adolescent Coping Scale (ACS) in CAMHS Terengganu
Research in stress among parents with adolescents having mental health problems
Research invalidation of Mental Health Questionnaire (MHQ) in child and adolescent
National research in stigma among children and adolescent with mental health (east coast
Module ‘Engaging the Adolescents’ – using HEAD55 framework (MOH group development)
Module ‘National Child Trauma Psychosocial Response Team Training’ (KKM, HELP university and UNICEF)
Module for Parenting skills for parents of children with mental health problems (MOH)
Assessment of resilience in adolescents (WHO & MOH development group
Strategic plan for Mental Health Service in Terengganu (Development group4
Suicide Prevention protocol for Terengganu
A pocket Guidebook of suicide prevention for paramedics in Terengganu
MOH My Health Portal website – development group for psychoeducation materials in child and adolescent mental health
Radio Malaysia Terengganu – invitation for psych °education slot in child and adolescent mental health and parenting
Psychoeducation pamphlet materials for parents, teachers and caregivers available at CAMHS

## Emerging ideas

CAMH service in Terengganu and the East coast of Malaysia has resulted in better skill reinforcement in areas of assessment and management of young people with mental health problems among medical practitioners and primary health care workers. Through receiving continuous funding and appropriate training, staff have achieved more confidence and are more able to care for their clients in their settings. This CAMHS establishment is believed to have an encouraging impact in the improvement of mental health among children, young people and their families in the East coast particularly Terengganu.

To date, there is no standardised service evaluation in our CAMHS to assess patient clinical and service outcome measurement that may indicate the impact of CAMHS to the population of Terengganu and also the east-coast. This standardised routine outcome measurement is still mostly unexplored not only in Terengganu but Malaysia in general. These future research findings may help us to provide useful information for improvement in the quality of treatment planning as well as the implementation of standardised outcome measurement for local and national benchmarking of CAMHS service in this country.

## Governance

National Institute of Health (NIH), Ministry of Health (MOH), Malaysia.

## Evidence of permissions to publish other people’s work

Application of approval for publications and presentations from Director General (DG) of Health Malaysia has been submitted to the NIH (Malaysia MOH).

## Disclosure statement

No potential conflict of interest was reported by the authors.

